# Comparison of short-term clinical outcomes between open-wedge high tibial osteotomy and tibial condylar valgus osteotomy

**DOI:** 10.1186/s12891-024-07205-7

**Published:** 2024-01-27

**Authors:** Takashi Higuchi, Hironobu Koseki, Akihiko Yonekura, Chieko Imai, Iku Tomonaga, Shinya Sunagawa, Umi Matsumura, Makoto Osaki

**Affiliations:** 1 Department of Physical Therapy, Osaka University of Human Sciences, Settsu, Japan; 2 Department of Health Sciences, Nagasaki University Graduate School of Biomedical Sciences, 1-7-1 Sakamoto, Nagasaki, 852-8520 Japan; 3 Institute of Biomedical Sciences, Nagasaki University, Nagasaki, Japan; 4 Department of Orthopedic Surgery, Nagasaki University Graduate School of Biomedical Sciences, Nagasaki, Japan

**Keywords:** Knee osteoarthritis, High tibial osteotomy, Tibial condylar valgus osteotomy

## Abstract

**Background:**

This study aimed to compare radiological features and short-term clinical outcomes between open-wedge high tibial osteotomy (OWHTO) and tibial condylar valgus osteotomy (TCVO), to provide information facilitating decision-making regarding those two procedures.

**Methods:**

Twenty-seven cases involving 30 knees that had undergone OWHTO (HTO group) and eighteen cases involving 19 knees that had undergone TCVO (TCVO group) for medial compartment knee osteoarthritis (OA) were retrospectively evaluated. Patient characteristics, severity of knee OA, lower limb alignment, joint congruity and instability were measured from standing full-length leg and knee radiographs obtained before and 1 year after surgery. Range of motion in the knee joint was measured and Knee Injury and Osteoarthritis Outcome Score (KOOS) was obtained to evaluate clinical results preoperatively and 1 year postoperatively.

**Results:**

Mean age was significantly higher in the TCVO group than in the HTO group. Radiological features in the TCVO group included greater frequencies of advanced knee OA, varus lower limb malalignment, higher joint line convergence angle, and varus-valgus joint instability compared to the HTO group before surgery. However, alignment of the lower limb and joint instability improved to comparable levels after surgery in both groups. Maximum flexion angles were significantly lower in the TCVO group than in the HTO group both pre- and postoperatively. Mean values in all KOOS subscales recovered similarly after surgery in both groups, although postoperative scores on three subscales (Symptom, Pain, and ADL) were lower in the TCVO group (Symptom: HTO, 79.0; TCVO, 67.5; Pain: HTO, 80.5; TCVO, 71.1; ADL: HTO, 86.9; TCVO, 78.0).

**Conclusions:**

Both osteotomy procedures improved short-term clinical outcomes postoperatively. TCVO appears preferable in cases of advanced knee OA with incongruity and high varus-valgus joint instability. An appropriate choice of osteotomy procedure is important to obtain favorable clinical outcomes.

## Background

Knee osteoarthritis (OA) is a progressive degenerative disease characterized by a gradual loss of articular cartilage around the knee, and is one of the most common musculoskeletal disorders, especially among the elderly [1–3]. Yoshimura et al. [4] reported that in Japan, symptomatic and asymptomatic knee OA affect approximately 7.8 million and 25.3 million individuals over 40 years old, respectively. Osteotomy procedures have been recommended for young and physically active knee OA patients wanting to maintain a wide range of motion (ROM), or for individuals who participate in high-demand activities and want to avoid prosthetic arthroplasty [5, 6]. A number of studies have reported favorable outcomes after osteotomies in the surgical treatment of medial unicompartmental knee OA [7–9]. High tibial osteotomy (HTO) is based on the concept of realignment to redistribute weight-bearing and mechanical stresses laterally to better preserved areas, relieving pain and improving function [9–11]. As tibiofibular joint disruption and peroneal nerve injury are potential complications associated with lateral closed-wedge HTO, open-wedge HTO (OWHTO) has gained popularity as a procedure utilizing a medial approach to avoid such complications [12–14]. Recent developments in internal fixator devices and surgical techniques have enabled early bone union and gap filling, contributing to better clinical outcomes [15, 16]. Even with OWHTO, however, risks include lateral hinge fracture (6–27%) [14, 17–19], delayed union or nonunion (9.2%) [14], and loss of correction (2.2–3.6%) [14, 20]. Concerns also remain about limited knee extension and disease progression due to ligamentous joint laxity [21, 22]. Recent studies have revealed that in severe knee OA with a high joint line convergence angle (JLCA), correction by HTO alone is not enough to restore normal joint geometry and biomechanics [23, 24]. Furthermore, a Kellgren–Lawrence (K/L) grade [25] ≥ 2 and laxity of the knee joint are thought to be risk factors in knee OA for declining clinical outcomes after HTO [22, 26]. Hence, in terms of indications, HTO is restricted to patients with mild to moderate medial knee OA in which high joint stability is maintained [6, 7].

Tibial condylar valgus osteotomy (TCVO) is an L-shaped osteotomy developed in the 1990s in Japan that also corrects lower extremity alignment from varus to valgus and shifts the weight-bearing (mechanical) axis laterally [27]. TCVO together with remodeling of the shape of the tibial plateau can improve femorotibial joint congruity and stability by levering up the medial tibial joint line. The combined features of osteotomy and arthroplasty thus appear promising for effective treatment of severe knee OA [28]. Due to improvements in implants over recent years, TCVO is now making use of locking plates, resulting in shorter postoperative rehabilitation. At our institute, OWHTO and TCVO are selected on a case-by-case basis for medial knee OA and have yielded almost entirely successful results [27]. However, TCVO is not widespread because of the technical difficulty and uncertainty regarding the universality of indication criteria.

The purpose of this study was to evaluate and compare short-term clinical outcomes between OWHTO and TCVO in detail, and to facilitate decision-making when choosing between the two surgical techniques.

## Methods

### Subjects

A total of 45 cases involving 49 knees that had undergone tibial osteotomy at our institute between December 2012 and December 2014 were retrospectively evaluated and included in this study. Indications for osteotomy were medial unicompartmental knee OA in relatively young patients (< 65 years old) and individuals participating in physically high-demand activities with a near-normal lateral femorotibial compartment, flexion ROM > 90° and flexion contracture < 10°. Patients with lateral unicompartmental knee OA, advanced patellofemoral arthritis, lateral bowing of the femur, inflammatory arthritis (such as rheumatoid arthritis), or current smoker status were excluded from osteotomy surgery. Middle-to-end-stage OA knees with depression- or convex-type (also called “pagoda deformity [24, 29]”) tibial plateau, lateral joint space widening (over 4–5° JLCA on the standing radiograph), and lateral tibial thrust > 1 cm were indicated for TCVO, whereas other cases such as flat-type tibial plateau were indicated for OWHTO, in accordance with the criteria of the International Society of Arthroscopy, Knee Surgery and Orthopedic Sports Medicine [6]. The HTO group comprised 27 cases (30 knees) that had undergone OWHTO. The TCVO group comprised 18 cases (19 knees) that had undergone TCVO. The present study was approved by the research ethics committee at Nagasaki University Graduate School of Biomedical Sciences (approval no. 2015–15082031), and all patients provided informed consent for participation and approval for their data to be published.

### Surgical procedures

The correction angle was estimated by preoperative planning using anteroposterior long-leg weight-bearing radiographs and finally determined by the alignment rod connecting the hip center to the ankle center intraoperatively, aiming to achieve 62% and 60% of the weight-bearing line in OWHTO and TCVO, respectively [16, 24, 30]. Although the threshold for mechanical medial proximal tibial angle (mMPTA) was set as < 95° for both osteotomies, predicting postoperative mMPTA in TCVO is difficult. The patient was placed in the supine position on a radiolucent operating table and a tourniquet was applied. Initial arthroscopy was performed to document medial-compartment arthritis and to assess the status of the lateral and patellofemoral compartments and menisci.

#### OWHTO

Biplanar open wedge osteotomy was performed as described by Staubli et al. [16]. A skin incision was made at the proximal tibia through the pes anserinus. Proximal to the pes anserinus, the medial collateral ligament (MCL) was dissected off the posteromedial cortex of the tibia and a blunt Hohmann retractor was inserted to protect the neurovascular structures. Two guide wires were inserted at a point 3.5–4 cm below the medial joint line and passing obliquely 1 cm below the lateral articular margin of the tibia towards the tip of the fibular head. The first osteotomy was performed distal to the guide wires to the upper position of the proximal tibiofibular joint. The osteotomy was incomplete, leaving intact 10 mm of lateral cortex (referred to as the bone bridge) to serve as a hinge point during opening of the osteotomy. The second frontal osteotomy plane started in the anterior one-third of the proximal tibia at an angle of 100° to the first osteotomy plane. An osteotomy was gradually opened until the desired, preoperatively determined alignment had been reached. After obtaining the planned gap, the osteotomized gap was filled with two triangular wedges of bone substitute comprising hydroxyapatite with beta-tricalcium phosphate (β-TCP) with 60% porosity (Osferion®; Olympus Terumo Biomaterials Corp., Tokyo, Japan). A TomoFix™ plate (DePuy Synthes, West Chester, PA) was placed on the anteromedial aspect of the tibia and locking head screws were inserted. After a temporary lag screw was inserted distal to the osteotomy, the distal locking head screws were inserted, then the lag screw was replaced by a locking head screw.

#### TCVO

**Fig. 1:**
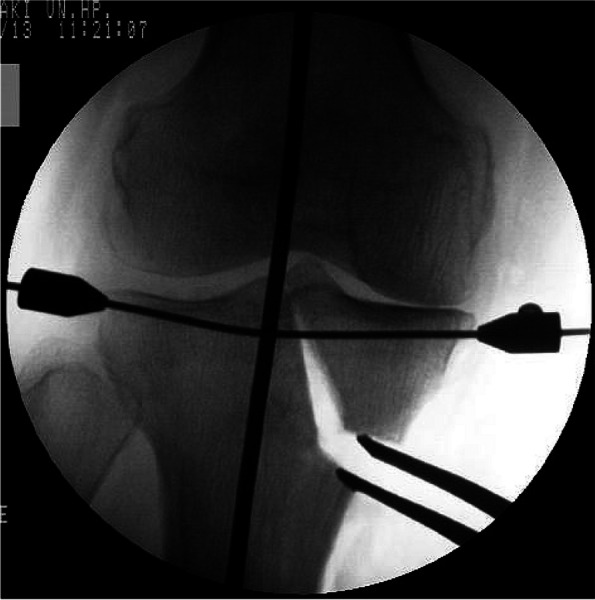
Intraoperative fluoroscopic image. Valgus correction is performed using the spreader. A Kirschner wire was inserted, and stoppers were attached to both ends to prevent separation and hinge instability of the tibial plateau before the valgus correction

**Fig. 2:**
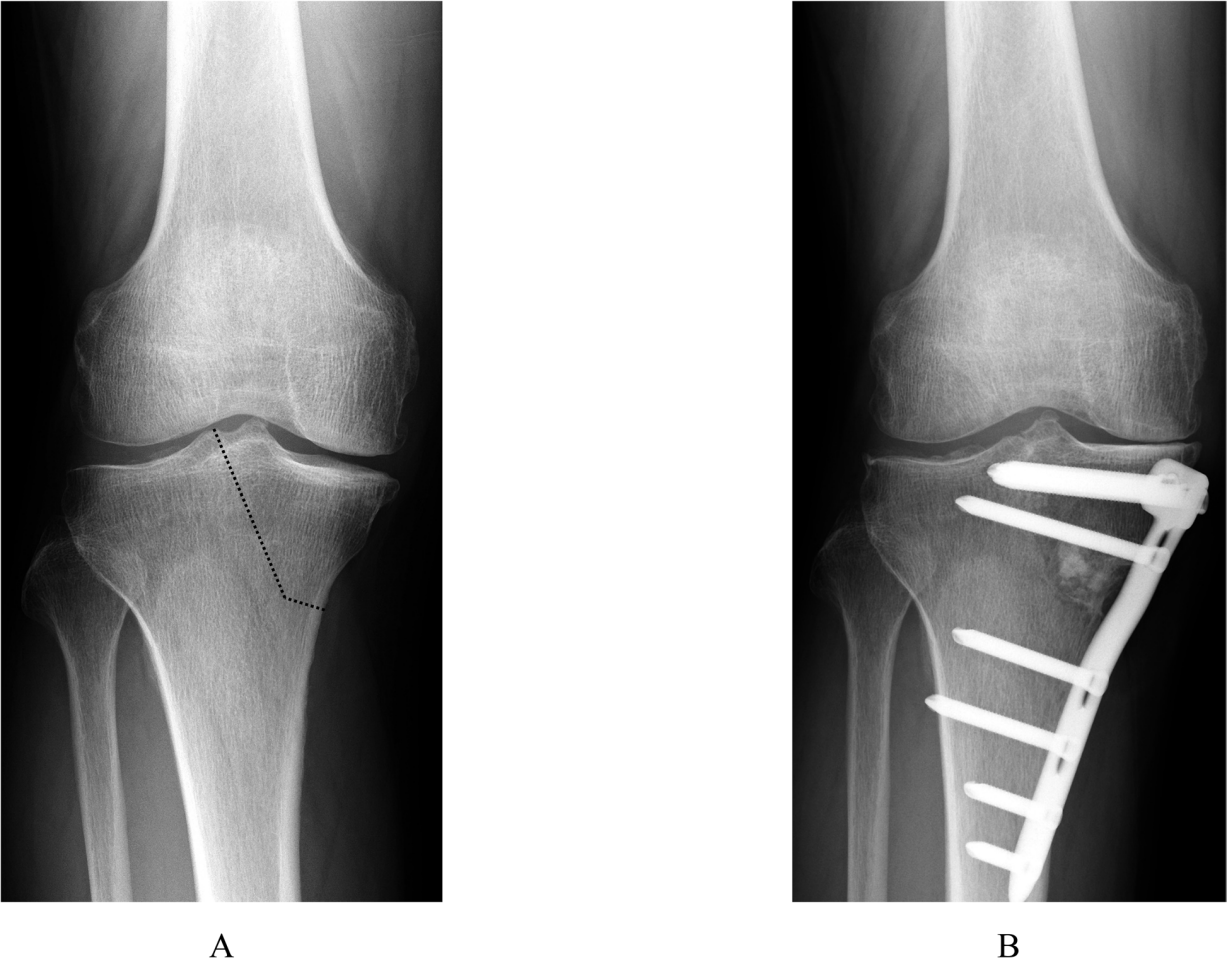
Anteroposterior radiographs of full-length legs in a standing position (**A**) before and (**B**) after TCVO. The L-shaped osteotomy is opened and fixed with a TomoFix™ plate. The opened space is filled with granular β-TCP

A curved skin incision was made from the point of the anteroposterior center and 1 cm distal through the medial joint line to the distal tibial tuberosity. Half of the proximal part of the pes anserinus and superficial layer of the MCL were dissected subperiosteally. The L-shaped osteotomy was implemented at the medial tibial tuberosity as the apex and extended towards the lateral intercondylar eminence vertically with a chisel, taking care not to injure any popliteal blood vessels. After the longitudinal cut, the transverse cut was performed from anteromedial to the tibial tubercle, using a bone saw and chisel. Mild valgus force was applied to the leg and completion of the osteotomy was confirmed on intraoperative radiographic imaging. A Kirschner wire was inserted, and stoppers were attached to both ends to prevent separation of the tibial plateau (Fig. 1). The osteotomy was opened with gradual valgus force until the desired, preoperatively determined alignment had been achieved. After this correction, a TomoFix™ plate was affixed to the anteromedial aspect of the tibia using locking screws. Granular β-TCP was used to fill the opened gap space (Fig. 2A, B).

### Radiological evaluations

Pre- and postoperative standardized anteroposterior radiographs of full-length legs in a standing position were taken with the feet in a neutral position. Radiographs of the knee joint and manual varus-valgus stress radiographs were also obtained and used for the following measurements.

**Fig. 3:**
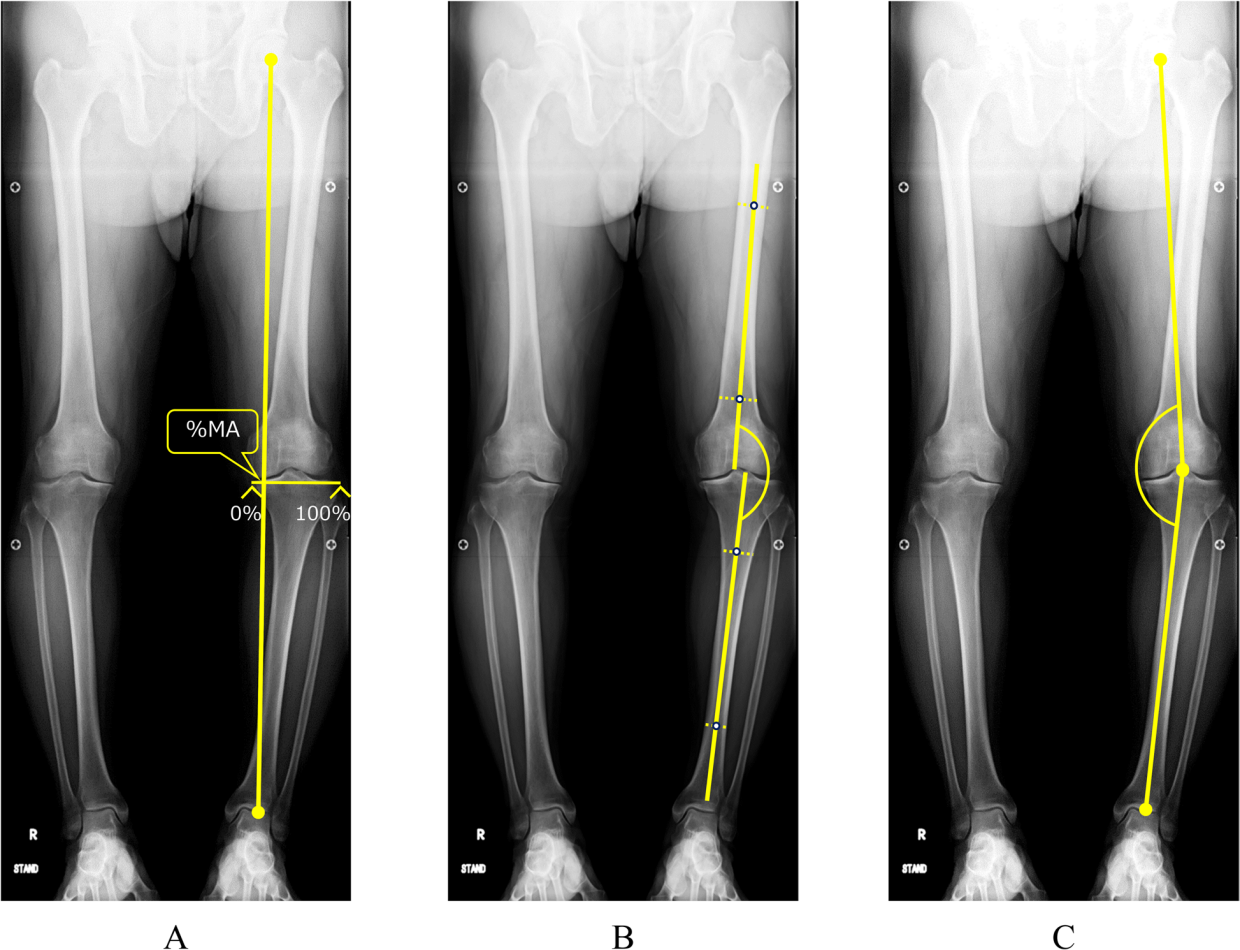
Percentage of the mechanical axis (%MA) (**A**), femorotibial angle (FTA) (**B**), and hip-knee-ankle angle (HKA angle) (**C**) are measured to evaluate leg alignment

K/L grade was used to classify the severity of knee OA. The mechanical axis (percentage of the mechanical axis: %MA), femorotibial angle (FTA), and hip-knee-ankle angle (HKA angle) were measured to evaluate lower limb alignment (Fig. 3A–C). The %MA indicates the point of intersection between the mechanical axis (a line drawn from the center of the femoral head to the center of the ankle) and the tibial plateau, converted to a percentage from medial edge (0%) to lateral edge (100%) [27, 31]. The JLCA was measured to evaluate congruity of the knee joint, as the angle formed between a line tangential to the distal femoral condyle and the tibial plateau. Varus and valgus instability was assessed using 100-N stress radiography (Telos stress device; Austin & Associates, Fallston, MD), and the total amplitude of varus- and valgus-stress angle was identified as the laxity angle. Three observers evaluated radiographs from each patient twice, at a minimum interval of 2 weeks. Intra-observer reliability was assessed based on evaluations by the first author. Inter-observer reliability was assessed based on evaluations between the first and second authors. Readers were blinded to the initial measurements, and mean values were taken as the measured values.

### Clinical evaluations

Operation time, intraoperative blood loss, hospitalization time, and time to acquisition of full weight-bearing were determined. Flexion and extension ROM was measured to the nearest 5° using a long-arm goniometer both preoperatively and at 1 year postoperatively. The Knee Injury and Osteoarthritis Outcome Score (KOOS) was used to evaluate clinical patient-based outcomes preoperatively and at 1 year postoperatively. The KOOS has 5 subscales: symptoms, pain, activities of daily living (ADL), sports and recreational function (Sports/Rec), and knee-related quality of life (QoL) [32]. Minimal clinically important difference (MCID) values were calculated to determine the effectiveness of both surgical procedures [33]. The MCID was generally considered as the smallest difference in score for which patients notice a real clinical improvement [34].

### Statistical analysis

Statistical analysis was performed using SPSS Statistics version 22 (IBM, Armonk, NY). Data were assessed for normality of the distribution using the Shapiro–Wilk test. The unpaired *t*-test or Mann–Whitney *U*-test was used for comparisons between groups. The Pearson χ^2^ test or Fisher’s exact probability test was used for nominal variables. Paired *t*-tests or Wilcoxon tests were used for comparisons between before and after surgery. Results were expressed as means and standard deviations. Values of *P* < 0.05 were considered statistically significant.

## Results

No cases of major or minor complications were observed, except for one instance of skin irritation in the HTO group. Background characteristics are shown in Table 1. Mean age was 59.5 ± 7.8 years for all patients, and the HTO group (57.8 ± 7.8 years) was significantly younger than the TCVO group (62.3 ± 7.0 years; *P* = 0.04). Other characteristics such as affected side, height, body weight, and BMI showed no significant differences between groups.

**Table 1:** Subject characteristics

	HTO	TCVO	*P*-value
Age (years)	57.8 ± 7.8	62.3 ± 7.0^a^	0.04
Gender (*n*)			
man/woman	15/15	11/8	0.59
Affected side (*n*)			
Right/Left	14/16	7/12	0.50
Height (cm)	161.6 ± 9.1	161.1 ± 8.4	0.97
Body weight (kg)	72.5 ± 14.2	73.7 ± 14.3	0.77
BMI (kg/m^2^)	27.7 ± 4.9	28.3 ± 4.2	0.53

^a^*P* < 0.05 compared to HTO group

**Fig. 4:**
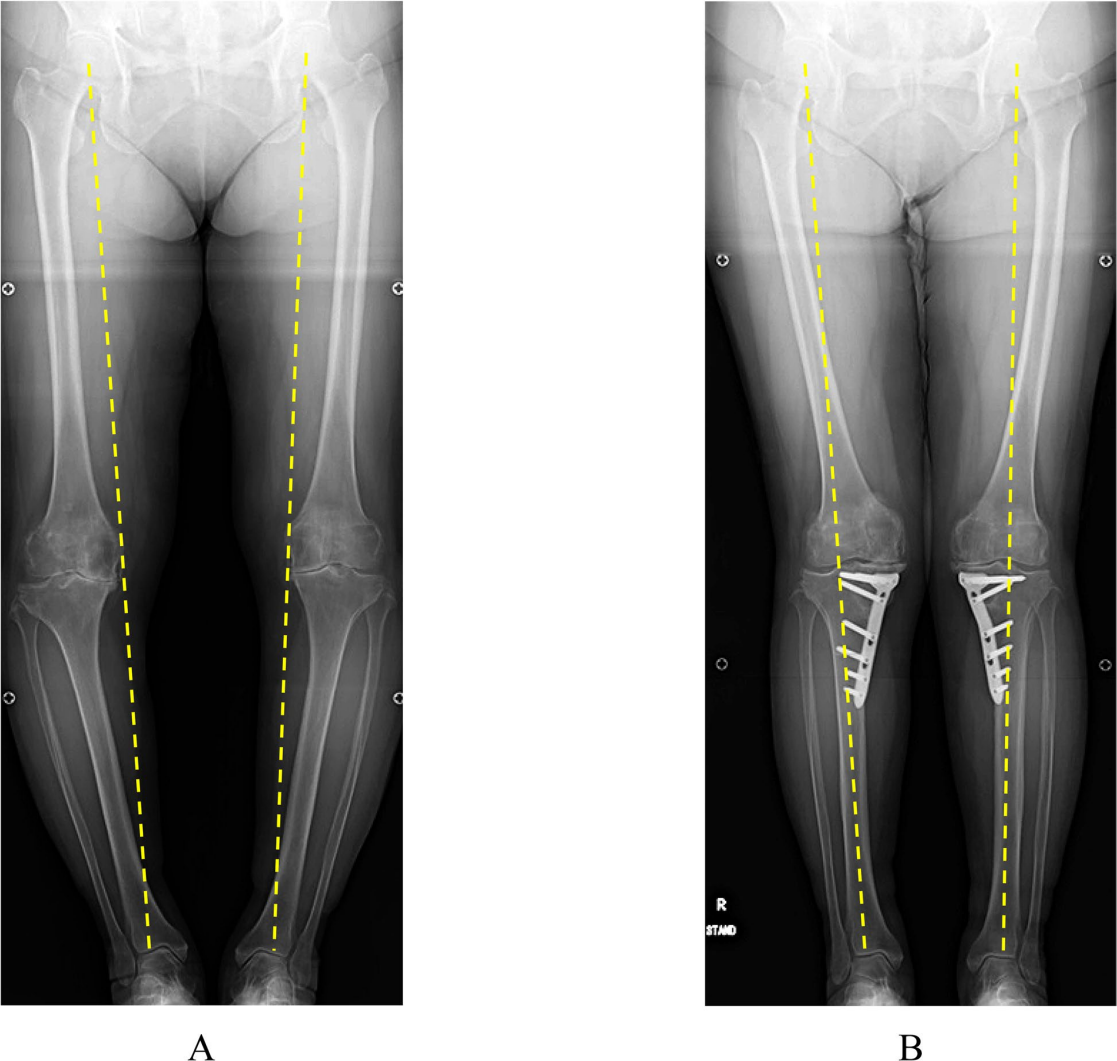
Pre- and postoperative radiographs with full-length legs in the standing position in a 63-year-old woman. Dotted line: mechanical axis (weight-bearing line). Preoperative: K/L grade (right 4, left 4), %MA (right -10%, left 0%). Postoperative: %MA (right 63%, left 59%)

Results for each radiological parameter, including joint congruity, instability, and ROM, are summarized in Table 2. Inter- and intra-observer reliabilities for radiographic parameters were all satisfactory. In terms of K/L grading, more advanced knee OA was more frequent in the TCVO group (grade 2 in 1 knee, grade 3 in 14 knees, grade 4 in 4 knees) than in the HTO group (grade 2 in 17 knees, grade 3 in 12 knees, grade 4 in 1 knee). Preoperative %MA was significantly lower in the TCVO group (10.8 ± 10.5%) than in the HTO group (21.6 ± 11.0%; *P* < 0.01). In terms of lower limb alignment before surgery, FTA was significantly higher in the TCVO group (183.7 ± 3.2°) than in the HTO group (180.3 ± 3.5°; *P* < 0.01) and HKA angle was significantly lower in the TCVO group (-9.0 ± 3.0°) than in the HTO group (-6.3 ± 2.2°; *P* < 0.01). In terms of pre- and postoperative comparisons in the HTO group, %MA and HKA were increased, whereas FTA was decreased significantly after surgery (*P* < 0.05). No significant differences in varus- or valgus-stress angles or laxity angle were seen between before and after surgery. In the TCVO group, lower limb alignment was improved in the same way as in the HTO group (Fig. 4A, B), while JLCA, varus and valgus stress angles, and laxity angle were significantly decreased after surgery (*P* < 0.05). Postoperative varus-stress angle was markedly declined relative to the HTO group. Preoperative ROM of the knee joint was slightly improved at 1 year after surgery in both groups. However, flexion angle was lower pre- and postoperatively in the TCVO group than in the HTO group. On the other hand, postoperative extension angle was significantly lower in the TCVO group than in the HTO group.

**Table 2:** Radiological parameters, instability, and ROM

	HTO		TCVO	
	Pre-op	Post-op	Pre-op	Post-op
K/L grade				
(II/III/IV)	17/12/1		1/14/4^a^	
%MA (%)	21.6 ± 11.0	64.7 ± 8.3^b^	10.8 ± 10.5^d^	59.8 ± 5.3^b^
FTA (°)	180.3 ± 3.5	169.1 ± 2.7^b^	183.7 ± 3.2^d^	170.9 ± 2.6^b,e^
HKA(°)	-6.3 ± 2.2	4.2 ± 2.2^b^	-9.0 ± 3.0^d^	3.5 ± 1.9^b^
Varus stress angle (°)	5.2 ± 0.9	6.6 ± 2.0	7.5 ± 1.4^d^	4.5 ± 1.8^b,e^
Valgus stress angle (°)	2.1 ± 2.1	0.9 ± 1.9	3.4 ± 2.3	1.2 ± 1.3^b^
Laxity angle (°)	7.0 ± 2.2	7.5 ± 3.1	10.9 ± 2.6^d^	5.6 ± 2.1^b^
JLCA (°)	1.4 ± 1.5	1.0 ± 0.9	4.9 ± 1.3^d^	0.8 ± 1.0^c^
ROM (°)				
Flexion	134.1 ± 10.2	137.6 ± 7.7	125.6 ± 13.0^d^	126.8 ± 10.3^e^
Extension	-2.0 ± 5.1	-1.4 ± 3.5	-3.8 ± 3.9	-4.1 ± 3.6^e^

^a^*P* < 0.01 compared to HTO

^b^*P* < 0.05 compared to preoperatively

^c^*P* < 0.01 compared to preoperatively

^d^*P* < 0.01 compared to pre-HTO

^e^*P* < 0.05 compared to post-HTO

Operation time was significantly longer in the TCVO group (170.2 ± 27.9 min) than in the HTO group (149.4 ± 37.1 min; *P* < 0.05). However, no significant differences were apparent in intraoperative blood loss, hospitalization time, or time to acquisition of full weight-bearing (Table 3). Results for each subscale of KOOS are shown in Table 4. In both groups, total score and all subscale scores improved significantly after surgery. Mean change in the KOOS was greater than the MCID in both groups. The Symptom, Pain, and ADL subscales of the KOOS at 1 year postoperatively were all significantly higher in the HTO group than in the TCVO group.

**Table 3:** Operation time, intraoperative blood loss, hospitalization time and time to acquisition of full weight-bearing

	HTO	TCVO	*P*-value
Operation time (min.)	149.4 ± 37.1	170.2 ± 27.9^a^	0.04
Intraoperative blood loss (g)	20.0 ± 17.0	29.7 ± 19.7	0.32
Hospitalization time (day)	8.6 ± 2.7	9.5 ± 2.4	0.28
Full weight-bearing (day)	35.0 ± 11.3	37.1 ± 11.3	0.63

*min* Minute

^a^*P* < 0.05 compared to HTO group

**Table 4:** Pre- and postoperative KOOS

	HTO			TCVO			MCID
	Pre-op	Post-op	amaount of change	Pre-op	Post-op	amount of change	
Symptom	57.9 ± 20.6	79.0 ± 14.6^a^	25.0 ± 22.0	53.8 ± 17.7	67.5 ± 13.1^a,b^	19.3 ± 23.7	15.4
Pain	49.9 ± 19.3	80.5 ± 12.2^a^	33.9 ± 21.9	44.4 ± 15.2	71.1 ± 14.6^a,b^	31.3 ± 22.4	17.0
ADL	66.1 ± 14.6	86.9 ± 10.5^a^	25.2 ± 20.6	58.0 ± 14.0	78.0 ± 11.9^a,b^	26.0 ± 20.7	15.1
Sports/Rec	30.4 ± 23.4	60.7 ± 23.4^a^	32.3 ± 28.0	19.4 ± 20.7	50.0 ± 20.5^a^	32.6 ± 22.8	11.2
QOL	27.5 ± 17.7	63.5 ± 21.5^a^	37.9 ± 20.3	30.5 ± 19.4	54.8 ± 19.2^a^	27.5 ± 24.3	16.5

*MCID *The Minimal Clinically Important Difference

^a^*P* < 0.01 compared to preoperatively

^b^*P* < 0.05 compared to post-HTO

## Discussion

The present results demonstrated that preoperative age, joint congruity and laxity, K/L grade, and FTA were higher and %MA and HKA angle were lower in the TCVO group than in the HTO group, and that short-term clinical outcomes including KOOS score were improved significantly by both osteotomy procedures. Factors such as older age, severity of knee OA, and joint laxity before surgery have been reported as causes of deteriorated clinical outcomes after HTO [26, 35]. Driban et al. [35] reported a negative correlation between age and clinical outcomes after HTO. Efe et al. [26] reported a K/L grade ≥ 3 as one factor associated with poorer clinical outcomes at an average of 9.6 years after HTO. Some other studies have also reported advanced knee OA and severe malalignment as contributors to HTO failure [7, 36, 37]. Only one previous study has reported satisfactory clinical outcomes from TCVO for K/L grade 3 or 4 patients, but the details were not described [27]. TCVO could thus be considered an effective surgical procedure for cases with more advanced knee OA and severe varus deformity. Mean %MA and FTA of the TCVO group in the present study were 10.8 ± 10.5% and 183.7 ± 3.2°, respectively. These results suggest that the alignment criteria of TCVO include a %MA of 5–15% and an FTA of 183–186°, as values for which clinical outcomes from HTO are thought to be declined.

Although HTO can reportedly improve stability of the knee joint [38, 39], chronic joint instability such as lateral thrust phenomenon remains a major factor affecting clinical outcomes. In addition, coronal plane joint incongruity and laxity have been reported as a cause of deteriorated clinical outcomes in HTO [22, 40, 41]. The removal of any torn medial meniscus may accelerate the progression of joint instability and knee OA [42, 43]. HTO with ligament reconstruction is one surgical option for the treatment of joint laxity [44–46], but requires greater surgical invasion and prolongs rehabilitation and hospitalization [46], in addition to increasing medical costs. TCVO together with remodeling of the shape of the tibial plateau can improve femorotibial joint congruity and stability. Increased tension in the cruciate ligaments by making the tibial plateau concave using the L-shaped osteotomy also contributes to better joint stability [28, 47]. Our results revealed that TCVO could reduce the mean JLCA from 4.9° to 0.8°, varus stress angle from 7.5° to 4.5°, and the laxity angle from 10.9° to 5.6°, without any ligament reconstruction. TCVO is thus desirable for knee OA involving higher joint laxity in the coronal plane.

Both flexion and extension ROM were restricted after TCVO compared to HTO. One possible explanation is that the severity of knee OA and age were higher in the TCVO group. Preoperative ROM is known to affect postoperative ROM. In addition, Naudie et al. [48] reported preoperative ROM < 120° as a cause of early failure of HTO. Another explanation is the tibial plateau morphology after TCVO. As mentioned above, TCVO remodels the tibial plateau to a concave shape, and this wedges the femoral condyle from both sides and increases tension in the cruciate ligaments [24, 28]. In addition, varus angle and joint laxity angle were significantly lower in the TCVO group than in the HTO group after surgery, although these values were also higher before surgery. TCVO thus has a positive aspect that increases joint stability, but the corollary is that ROM could be restricted, particularly during extension.

In TCVO, great care must be taken during L-shaped osteotomy to prevent popliteal blood vessels or to avoid cutting beyond the osteotomy line. Moreover, the locking plate sometimes needs to be bent depending on the shape of the proximal tibia. These factors might contribute the longer operation time in TCVO group compared to that in HTO group. In our study, no significant difference was found in intraoperative blood loss, hospitalization time, and time to acquisition of full weight-bearing. Recently, Full wight-bearing is allowed 1 week after surgery in both procedures, depending on the severity of pain. Therefore, time to acquisition of full weight-bearing is earlier than the result of this study.

In both groups, the KOOS score was significantly improved, and the amount of change exceeded the MCID of the KOOS. Patients in the present study were relatively older than in previous research [49–52], so our criteria for each surgical procedure were considered appropriate. However, KOOS score after surgery was overall lower in the TCVO group than in the HTO group, and scores for the Symptom, Pain, and ADL subscales in the TCVO group were significantly lower. The lower KOOS score preoperatively, older age, and the fact that about 90% of patients in the TCVO group had severe knee OA with high joint instability were considered causes of the lower postoperative KOOS score.

Based on the present results, the advantages of TCVO appear to be: 1) correction of varus malalignment of the lower extremity; 2) reconstruction of medial articular deformation of the tibial plateau; and 3) reduction of joint incongruity and laxity. The following advantageous points have also been indicated: 4) early weight-bearing because the osteotomy line does not reach the lateral tibial condyle; 5) low risk of hinge fracture; and 6) reduction of subluxated lateral joints during the operation compared to HTO [27, 53, 54]. TCVO is thought to represent an effective surgical procedure for patients with advanced varus knee OA, inclined medial tibial plateau, widened lateral femorotibial joint, and high joint instability. However, attention must be paid to the disadvantages of TCVO. First, correction of the tibia to a valgus position is limited only to the angle at which the lateral joint is reduced. Prudent preoperative planning is required to compare correctable and estimated postoperative %MA. When under-correction is anticipated in preoperative planning, due to femoral deformity (such as mechanical lateral distal femoral angle ≥ 90°) or less instability (such as laxity angle < correction angle), additional OWHTO and/or distal femoral osteotomy would be needed to achieve optimal alignment and anatomical joint-line obliquity [24, 53, 55]. Second, soft-tissue balance cannot be modified directly by this procedure.

Limitations in this study included the small number of cases and short duration of follow-up (1 year after surgery). Lee et al. [56] reported barely any correction loss from 1 year after HTO, but radiographic changes (such as progression of knee OA and correction loss) must be pursued over the long term after surgery. TCVO with a locking plate and minimally invasive surgical techniques have been introduced since 2008. In addition, the possibility of selection bias from the indication criteria for each procedure could not be ruled out. Further study is therefore warranted to include a large sample size and a prospective design is needed to better clarify the exact indications and determine the clinical availability of TCVO. Recently, obesity and insufficient exercise have become increasingly prevalent among young adults, raising concerns that the age of onset for knee OA might fall. In the future, demands for osteotomy seem likely to rise as regenerative treatments for articular cartilage or meniscus become widespread. As osteotomy is much more cost-effective than total knee arthroplasty (TKA) [57], the present value of TCVO will be increased as a surgical alternative to TKA in the treatment of knee OA.

## Conclusion

We compared short-term clinical outcomes between OWHTO and TCVO. Both procedures improved %MA, lower limb alignment, and KOOS, and maintained ROM. An understanding of the concepts, advantages, and disadvantages of OWHTO and TCVO is important for selection of the appropriate procedure according to individual pathological conditions such as lower limb alignment and joint stability.

## Data Availability

The datasets used and analyzed during the current study are available from the corresponding author on reasonable request.

## Abbreviations

OA: Osteoarthritis
ROM: Range of motion
HTO: High tibial osteotomy
OWHTO: Open-wedge HTO
JLCA: Joint line convergence angle
K/L: Kellgren–Lawrence
TCVO: Tibial condylar valgus osteotomy
mMPTA: Mechanical medial proxximal tibial angle
MCL: Medial collateral ligament
β-TCP: Beta-tricalcium phosphate
%MA: Percentage of the mechanical axis
FTA: Femorotibial angle
HKA angle: Hip-knee-ankle angle
KOOS: Knee Injury and Osteoarthritis Outcome Score
ADL: Activities of daily living
Sports/Rec: Sports and Recreational function
QoL: Quality of life
MCID: Minimal Clinically Important Difference
TKA: Total knee arthroplasty
